# EEG-Based Detection of Braking Intention Under Different Car Driving Conditions

**DOI:** 10.3389/fninf.2018.00029

**Published:** 2018-05-29

**Authors:** Luis G. Hernández, Oscar Martinez Mozos, José M. Ferrández, Javier M. Antelis

**Affiliations:** ^1^Tecnologico de Monterrey, Escuela de Ingeniería y Ciencias, Zapopan, Mexico; ^2^DETCP, Technical University of Cartagena, Cartagena, Spain

**Keywords:** driving, braking, intention, electroencephalogram, detection, stress, workload, fatigue

## Abstract

The anticipatory recognition of braking is essential to prevent traffic accidents. For instance, driving assistance systems can be useful to properly respond to emergency braking situations. Moreover, the response time to emergency braking situations can be affected and even increased by different driver's cognitive states caused by stress, fatigue, and extra workload. This work investigates the detection of emergency braking from driver's electroencephalographic (EEG) signals that precede the brake pedal actuation. Bioelectrical signals were recorded while participants were driving in a car simulator while avoiding potential collisions by performing emergency braking. In addition, participants were subjected to stress, workload, and fatigue. EEG signals were classified using support vector machines (SVM) and convolutional neural networks (CNN) in order to discriminate between braking intention and normal driving. Results showed significant recognition of emergency braking intention which was on average 71.1% for SVM and 71.8% CNN. In addition, the classification accuracy for the best participant was 80.1 and 88.1% for SVM and CNN, respectively. These results show the feasibility of incorporating recognizable driver's bioelectrical responses into advanced driver-assistance systems to carry out early detection of emergency braking situations which could be useful to reduce car accidents.

## 1. Introduction

According to the World Health Organization (WHO), every year traffic accidents cause the death of 1.3 million people around the world, additionally, about 50 million people suffer from a disability caused by accidents related to cars (WHO, 2011). By 2020, it is estimated that traffic accidents will be the fifth leading cause of death in the world, reaching 2.4 million deaths per year (WHO, 2013). Among the principal causes of the high car-related accidents and mortality are human errors (Subramanian, 2007) which are largely correlated to distractions, tiredness, or the simultaneous realization of other activities during driving (Allnutt, 1987; Horowitz and Dingus, 1992; Summala and Mikkola, 1994; Petridou and Moustaki, 2000). To mitigate this problem, driving assistance systems appeared as in-car technologies that aim to help and complement the human-based car-control in order to prevent potential accidents (National Highway Traffic Safety Administration, 2005). These systems employ internal sensors, like for example speedometers, accelerometers, and pedals level; and external sensors such as lidar, sonar, and video-cameras to obtain and to analyze information from the vehicle (e.g., orientation and twists) and from its surroundings (e.g., the presence of other cars or pedestrians, the road and whether conditions) that is used to help drivers to recognize and to react to potentially dangerous situations (Shaout et al., 2011; Smirnov and Lashkov, 2015). For instance, driving assistance systems can be useful to properly respond to emergency braking situations as those required by the sudden and unforeseen appearance of cars, bicycles, or persons. In these situations, if the external sensors recognize a potential upcoming crash and the internal sensors detect an abrupt and rapid activation of the brake pedal, then safety actions such as to speed-up the braking along with a proper maneuvering might be carried out. This procedure may generate a faster and controlled braking response than the one made by the driver alone, thus possibly preventing a potential accident.

Current driving assistance systems do not use driver's information such as eye or head movements, hand sweating, or hand pressure on the steering wheel, although this information might be correlated to fatigue or drowsiness (Liu et al., 2009; Li et al., 2017b), and thus can critically affect driving performance. In consequence, these systems might not respond adequately to dangerous situations caused by the driver's behavior (Janssen, 2001). To address those problems, recent works have proposed to employ information obtained from the driver (Paul et al., 2016). For example, driver's face images has been used to recognize distractions (Sigari et al., 2013; Fernández et al., 2016) or drowsiness (Liu and Salvucci, 2001) while physiological activity has been employed to detect fatigue (Li et al., 2017a) or drowsiness (Sahayadhas et al., 2012; lan Chen et al., 2015). The final aim in these approaches is to detect potential danger situations originated by human errors while driving (Janssen, 2001). In the case of emergency braking situations, the mechanical activation of the brake pedal is the final outcome in a series of cognitive and peripheral processes that include visual perception, mental assessment, motor planning, motor execution, and proprioceptive feedback (Sherk and Fowler, 2001; Saffarian et al., 2015). Hence, the brake pedal deflection, as any other human action, is preceded by cognitive processes that are, to some extent, observable in the central nervous system through the ongoing brain activity. Therefore, it seems feasible to study the driver's brain activity that precedes the moment at which the brake pedal is activated, in order to perform an early detection of emergency braking situations. This can help to reduce the braking reaction time and to prevent potential accidents. For instance, driving assistance systems can be useful to properly respond faster to emergency braking situations required by the sudden appearance of other agents on the road.

Recent research have addressed the detection of braking intention using non-invasive electroencephalographic (EEG) signals. The pioneer work of Haufe et al. (2011) showed that event-related potentials (ERP) recorded in a simulated driving environment can be used to distinguish emergency braking intention from non-braking driving. This study was then replicated in a real driving environment and their results confirmed the recognition of emergency braking prior to the mechanical activation of the brake pedal (Haufe et al., 2014). Subsequently, Kim et al. (2015) studied the brain electrical activity in diverse braking situations (soft, abrupt, and emergency) during simulated driving and their results showed neuronal correlations, in particular movement-related potentials (MRP) and event-related desynchronization (ERD), that can be used to distinguish between different types of braking intentions. The works of Chavarriaga et al. (2013) and Teng and Bi (2017) also studied and analyzed EEG signals for the early detection of various emergency braking situations. However, the previous works were carried out in fully controlled settings for the participants where they did not experience cognitive factors such as stress, workload, and fatigue that may affect or delay the execution of braking (da Silva, 2014; Paxion et al., 2014; Zhang and Kumada, 2017). This is critical because during real driving, drivers are commonly exposed to a combination of different factors that may interfere attention and decision making (Bouchner et al., 2009; Schweizer et al., 2013). Indeed, scientific evidence has shown that the attention level during the driving task is influenced by stress, workload, and fatigue, and thus they tend to increase the braking reaction time (Baulk et al., 2001; Lal and Craig, 2002; Wester et al., 2008; Borghini et al., 2014). Consequently, it is important to study the driver's brain activity during emergency braking when the level of attention is affected by the aforementioned cognitive factors.

This work proposes the detection of emergency braking intention using driver's electroencephalographic (EEG) brain signals recorded in a simulated driving environment that includes absence and presence of stress, workload, and fatigue. The rationale is to include these cognitive factors that may affect and delay the emergency braking response. In the experimental task, participants had to naturally drive a vehicle and to perform unexpected emergency braking to avoid crashing with another vehicle. This task was repeated under different cognitive situations including combinations of stress, workload, and fatigue. EEG signals from the driver were recorded in order to evaluate the recognition between braking intention and normal driving through a conventional classifier as support vector machines (SVM) and a novel classifiers model as convolutional neural networks (CNN). In recent studies, CNN models was used to classify mental tasks from EEG signals in a controlled experiment with successful results (Ren and Wu, 2014; Schirrmeister et al., 2017; Tabar and Halici, 2017). Therefore, these works are a a starting point for exploring the performance of a CNN model for classifying mental tasks in more complex environments, such as car driving. Our results show a significant detection rate of 71.1 and 71.8% for SVM and CNN, respectively, while the classification accuracy for the best participant was 80.1 and 88.1%. These results shows the feasibility of incorporating the driver's bio-electrical signals into driving assistance systems for the early detection of potential situations that might require emergency braking in order to reduce the accident and mortality associated with vehicular traffic. The rest of the paper is organized as follows. Section 2 describes the experimental set-up and the data analysis methodology; section 3 presents and discusses the experimental results and section 4 presents the conclusions.

## 2. Materials and methods

### 2.1. Participants

Seven right-handed male students (age range 20–26 years old) from our faculty voluntarily participated in this study. All participants approved the following inclusion criteria: (*i*) to know how to drive a vehicle either with manual or automatic clutch; (*ii*) to have a valid driver's license; (*iii*) to have no medical history of neurological and/or psychiatric diseases; (*iv*) to have normal or corrected-to-normal vision. The protocol was approved by the Comité de Ética en Investigación de la Escuela de Medicina del Instituto Tecnológico y de Estudios Superiores de Monterrey and the Comité de Investigación de la Escuela de Medicina del Instituto Tecnológico y de Estudios Superiores de Monterrey. All subjects gave written informed consent in accordance with the Declaration of Helsinki and they were duly informed about the goals of the research.

### 2.2. Driving system and environment

**Figure 1:**
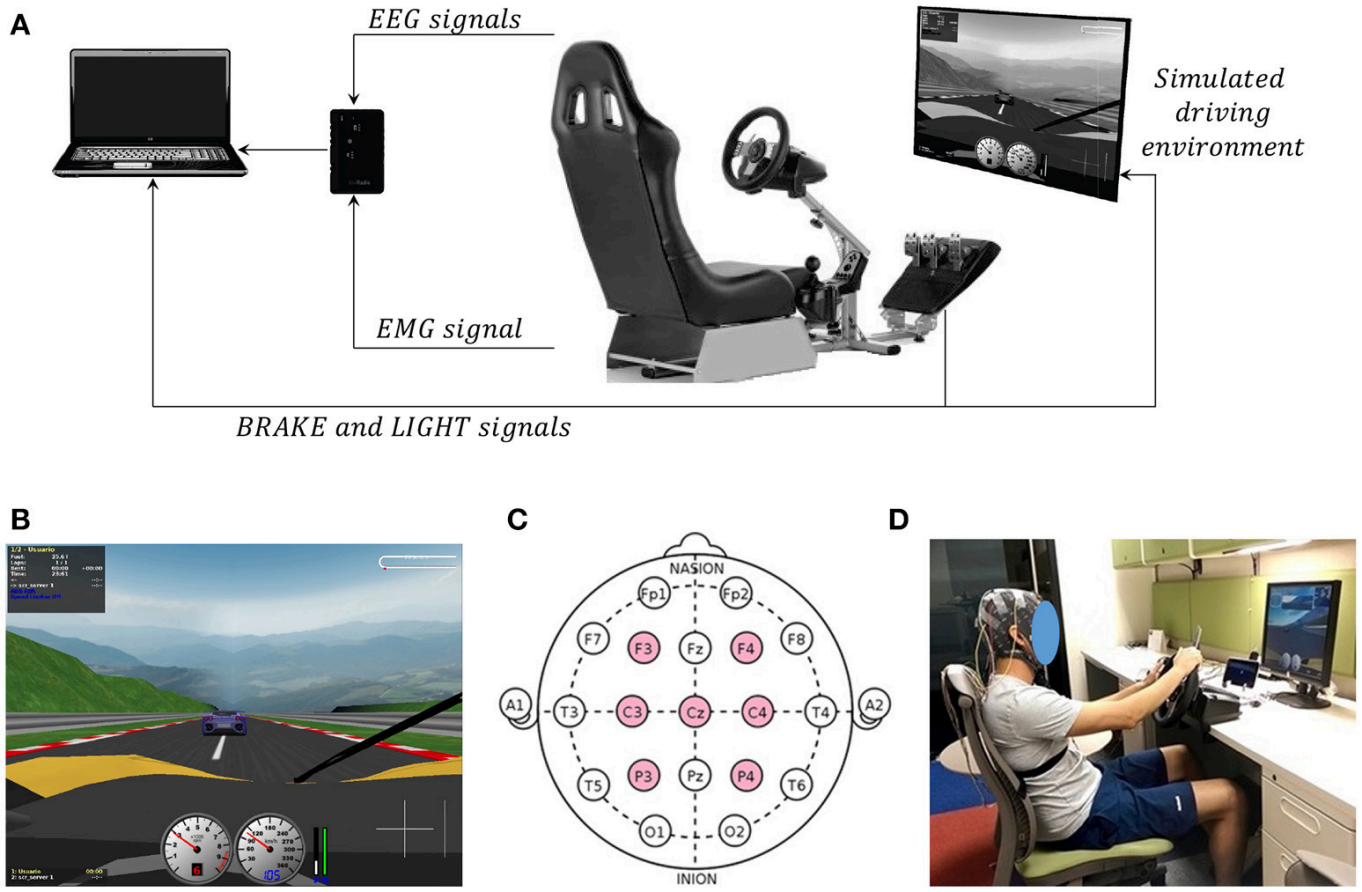
**(A)** Illustration of the driving system and environment. The driving system consisted of a commercial set of pedals, steering wheel and gear lever for driving simulators, a 19 inches flat-screen and a computer to control the execution of the experiment. The driving environment consisted of an oval track and contained the participant's vehicle and a guide vehicle. **(B)** Image of driving environment in a first person perspective as seen by the participants. **(C)** EEG electrode locations used in this experiment. **(D)** Snapshot of the experiment with a participant wearing the EEG electrodes (the participant gave written informed consent to publish this picture).

The driving system consisted of a set of gas, brake and clutch pedals, a steering wheel and a gear lever (G27 Logitech Racing Wheel) assembled in a car simulator rack, a 19 inches flat-screen to visualize the driving environment and a personal computer to manage and control the execution of the experiment and the acquisition of signals (see Figure 1A). The driving environment was developed using the open source software The Open Racing Car Simulator (TORCS) (Wymann et al., 2014) and consisted of a two-lane oval track of 3700 m, the participant's vehicle and a guide vehicle. This environment also contain other computer-controlled vehicles that did not interfere with the participant's and guide vehicles. The participant's vehicle is displayed in a first person perspective (see Figure 1B), has an automatic clutch and is completely controlled by the steering wheel and the pedals (the gear lever has not effect in the vehicle). The guide vehicle is a computer-controlled car that is displayed at the lane ahead of the participant's vehicle. Two vehicular signals were recorded from the driving system and environment: (*i*) the state of the guide vehicle rear brake lights or simply “LIGHT” (digital signal where a low level corresponds to lights off and a high level corresponds to lights on); (*ii*) the brake pedal level of the participant's vehicle or simply “BRAKE” (signal in the range of 0 to 1 with a resolution of 0.01, where 0 corresponds to no deflection while 1 corresponds to fully deflection of the pedal). These vehicular signals were recorded at a sampling frequency of 50 Hz.

### 2.3. Bio-electrical signals

Electroencephalographic (EEG) and electromyographic (EMG) activity was recorded during the execution of the experiment. These bio-electrical signals were acquired, amplified, and digitalized using the 8-channels wearable device for recording of human physiological signals BIORADIO PG (Great Lakes NeuroTech, USA). Seven EEG signals were recorded from the frontal, central, and parietal lobe (F3, F4, C3, C4, Cz, P3, P4) in accordance to the 10/10 international system (see Figure 1C). The ground and the reference electrodes were placed above the mastoid process in the right and left part of the head, respectively. Gold cup electrodes were used and conductive gel was applied to ensure impedance less than 5 KΩ. An EMG signal was recorded from the anterior tibial muscle from the right leg using a monopolar montage. The ground and reference were the same as for the EEG signals. A surface disposable electrode was used and the impedance was kept below 20 KΩ. EEG and EMG signals were recorded at a sampling frequency of 500 Hz and no filtering was applied.

### 2.4. Description of the experiment

Participants were seated in the car seat in front of the pedals, steering wheel, gear lever, and the computer screen (see Figure 1D). The experimental task was to drive the participant's vehicle while following the guide vehicle at a constant and fixed distance of ~10 m. To illustrate this distance to the participants, at the beginning of each experimental session the vehicles were stopped and separated by 10 m. The guide vehicle drives autonomously at a constant speed of 100 km/h and performs unexpected and sudden breaks up to reach a speed of 60 km/h. These braking actions are accompanied by the switching-on of the rear brake lights providing a visual stimulus to the participants that indicates to perform a response to avoid collision, i.e., to press the brake pedal. After 3 s, the guide vehicle accelerates gradually until reaching again 100 km/h and the participant must drive his vehicle while maintaining a distance of ~10 m. In case of collision, participants were instructed to interpret it as a normal circumstance with no effect on their driving performance or on the recorded data. Also, after a collision the experimental session is restarted normally. This emergency braking situation is repeatedly performed at variable occurrence intervals as follows: After performing an emergency braking situation, the guide vehicle was programmed to produce a new emergency braking situation in a pseudo-random time between [5−20] s after reaching again the speed of 100 km/h. That time interval was contemplated so that emergency braking is carried out between the speeds mentioned in the description of experiment (from 100 to 60 km/h) and that participants do not anticipate and generate an early braking response.

During the execution of the experiments, participants were exposed to different combinations with absence or presence of stress, workload, and fatigue. Stress was induced with an ambulance siren sound at an intensity of 90 dB which is within the range of discomfort for the human hearing (70–100 dB) (Chepesiuk, 2005). This external, repetitive and high annoying sound generates perturbation and anxiety to drivers leading to stress episodes (Wester et al., 2008). Workload was induced with a simultaneous attention task (SAT) that had to be performed while driving (Borghini et al., 2012; Maglione et al., 2014). The SAT consisted in touching the gear lever with the right hand (which has no effect in the driving of the vehicle) as a response to the presentation of an image with the symbol “X.” This image was randomly presented in a secondary 7 inches screen located in front of the participant without obstructing the field of view toward the main screen. Finally, fatigue was induced by considering the natural physical and mental exhaustion associated with the performance of daily life activities during the course of the day. Therefore, experiments were carried out during a day in the morning (between 09:00 and 12:00, where it is assumed that the participant is relaxed and rested because of the recent night's sleep) and in the afternoon (between 16:00 and 19:00, where it is assumed that the participant is tired because of the daily activities) (Baulk et al., 2001; Horne and Baulk, 2004; Komada et al., 2013). In order to keep natural physical and mental exhaustion, the participants were asked to sleep at least 7 h of restful sleep the night before the experiment, they were asked not to sleep during the day of the experiment, not drink coffee or other energy drinks and they were told not to smoke during the duration of all the experiments. Furthermore, the participants were questioned about their levels of fatigue before and after the execution of the experiment through the NASA Task Load Index (NASA-TLX) (Hart and Staveland, 1988). All eight possible combinations with absence or presence of stress, workload and fatigue were considered during the execution of emergency braking during the experiments. These experimental combinations are presented in Table 1.

**Table 1:** Description of the eight experimental combinations with absence (–) or presence (✓) of stress, workload and fatigue that were considered during the realization of emergency braking.

**Experimental combination**	**Stress**	**Workload**	**Fatigue**
*C*_*o*_	–	–	–
*C*_*s*_	✓	–	–
*C*_*w*_	–	✓	–
*C*_*f*_	–	–	✓
*C*_*s*+*w*_	✓	✓	–
*C*_*s*+*f*_	✓	–	✓
*C*_*w*+*f*_	–	✓	✓
*C*_*s*+*w*+*f*_	✓	✓	✓

*Combination C_o_ does not include any factor. Combinations C_s_, C_w_, and C_f_ only include stress, workload, and fatigue, respectively. Combinations C_s+w_, C_s+f_, C_w+f_, and C_s+w+f_ include two or three factors*.

**Figure 2:**
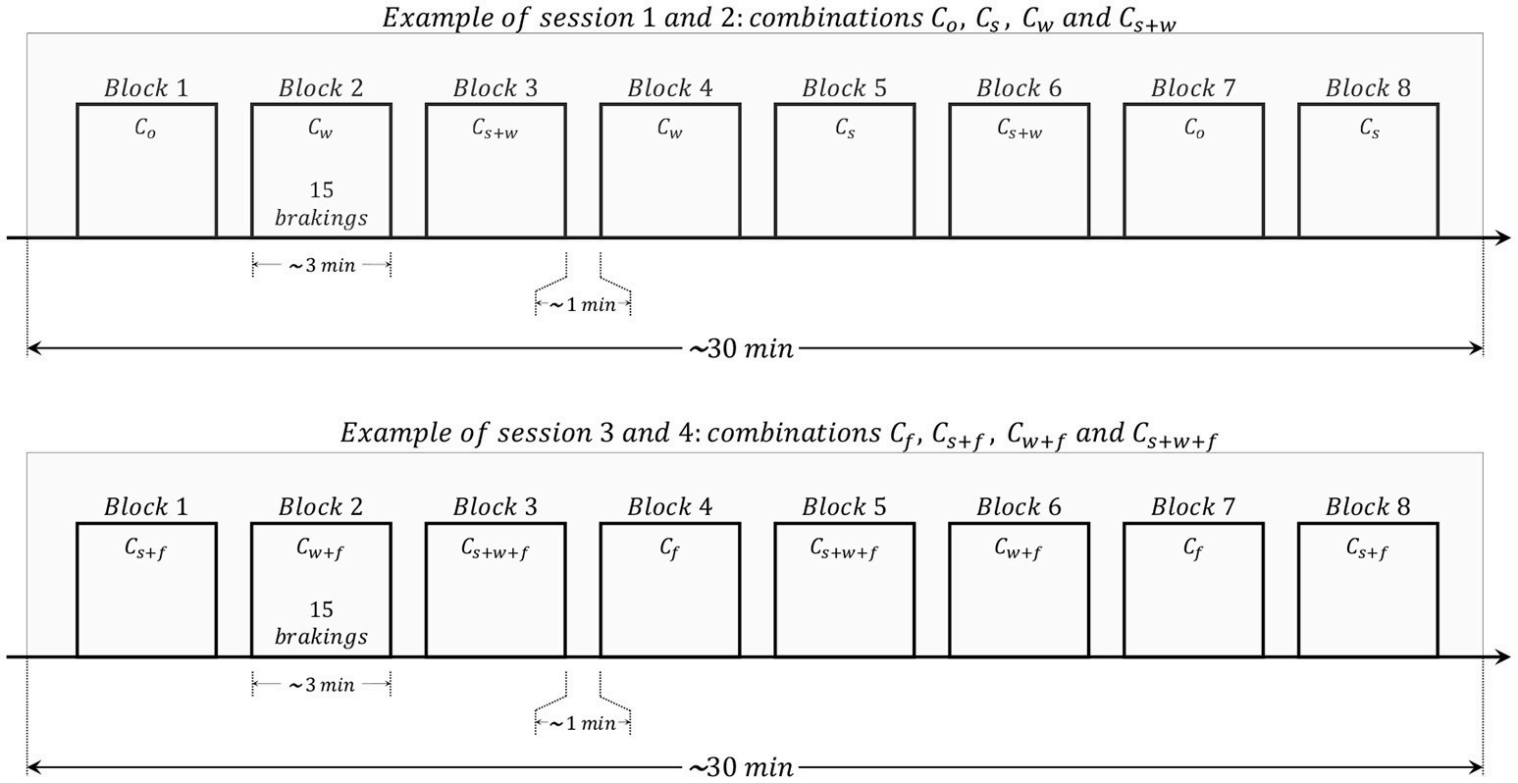
Illustration of the temporal sequence of a morning session **(Top)** and an afternoon session **(Bottom)**. Each session consisted of eight blocks. Each block contained 15 emergency braking of the same experimental combination. The experiment was carried out in four sessions (two in the morning and two in the afternoon). In total, 120 emergency braking situations were presented in each session yielding to a total of 480 emergency braking situations per participant.

The experiment was carried out in four sessions (two in the morning and two in the afternoon) of ~30 min each where the participant had to drive continuously. In each session, 120 emergency braking situations were presented, therefore, 480 emergency braking situations were recorded in total per participant. The rest period between sessions was ~10 min. Each session was composed of eight blocks and each block contained 15 emergency braking situations from the same experimental combination. Each block had a duration of ~3 min while the separation between blocks was ~1 min. To avoid habituation, the order of blocks in each session was pseudo-random. Figure 2 illustrates the temporal sequence of one morning session and one afternoon session. The two morning sessions contain blocks with emergency braking situations that correspond to experimental combinations *C*_*o*_, *C*_*s*_, *C*_*w*_, and *C*_*s*+*w*_ while the two afternoon sessions contain blocks with emergency braking that correspond to experimental combinations *C*_*f*_, *C*_*s*+*f*_, *C*_*w*+*f*_, and *C*_*s*+*w*+*f*_.

### 2.5. Preprocessing

**Figure 3:**
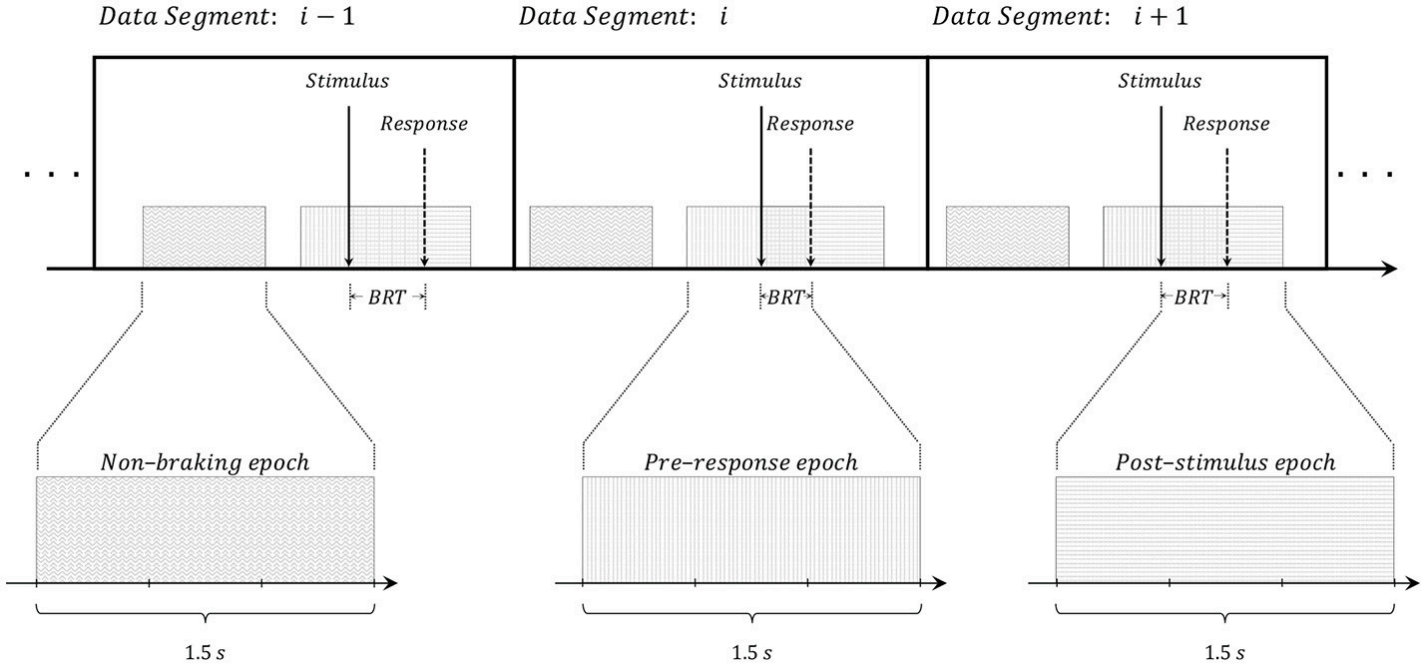
Graphical illustration of the data segments and the three types of epochs extracted from each of them: (*i*) Non-braking epochs: signals of 1.5 s that are more than 3 s apart from any stimulus and/or response (data without emergency braking); (*ii*) pre-response epochs: signals in the time interval [−1.5, 0] s where the reference *t* = 0 corresponds to the response (the first notable deflection of the participant's vehicle brake pedal); (*iii*) post-stimulus epochs: signals in the time interval [0, 1.5] s where the reference *t* = 0 corresponds to the stimulus (the guide vehicle switching-on of the rear brake lights).

LIGHT and BRAKE signals were upsampled while EEG and EMG signals were downsampled, in both cases to a sampling rate of 250 Hz. EEG signals were lowpass filtered at a cutoff frequency of 45 Hz using a 2nd-order zero-phase shift Chebychev-type filter and then common average referenced (CAR). EMG signal was bandpass-filtered from 1 to 90 Hz using a 2nd-order Chebychev-type filter and stop-band filtered at 60 Hz to reduce power line interference. The shift from 0 to 1 of the LIGHT signal was used as reference to identify the time instant of each stimulus (i.e., the time where the rear brake lights of the guide vehicle turned on) while the first post-stimulus time for which BRAKE ≥ 0.01 was used as reference to identify time instant of each response (i.e., the time of the first notable deflection of the brake pedal after a stimulus had occurred). The time instants of all stimuli were used as reference to trim the signals into consecutive data segments that span up to 2 s after the stimulus (see Figure 3). Thus, each data segment contains an emergency braking and includes the response (this was verified in the data analysis which showed responses lower than 2 s). Each data segment underwent visual inspection and those with incongruent vehicular signals (without post-stimulus activation of the brake pedal), incongruent EMG signals (without post-stimulus amplitude increasing), and noisy EEG signals (contaminated with muscle or eye artifacts) were discharged and not used in the rest of the study. As a result, the number of data segments across all participants was on average 428 ± 56 (minimum 300 and maximum 474).

For each data segment, three epochs of 1.5 s were extracted (Figure 3): (*i*) non-braking epochs or normal driving: signals of 1.5 s that are more than 3 s apart from any stimulus and/or response. These epochs do not include braking situations at all and do not overlap with post-stimulus or pre-response epochs. (*ii*) pre-response epochs: signals in the time interval [−1.5, 0] s where the reference *t* = 0 corresponds to the response. These epochs contain information that exclusively precede the deflection of the brake pedal; (*iii*) post-stimulus epochs: signals in the time interval [0, 1.5] s where the reference *t* = 0 corresponds to the stimulus. These epochs contain information immediately subsequent to the switching-on of the rear brake lights; This procedure resulted in three different datasets. The dataset of post-stimulus epochs were employed to study the emergency braking situations while the datasets of pre-response and normal driving epochs were used to distinguish emergency braking intention from normal driving using brain signals.

### 2.6. Data analysis

To study the emergency braking situations, the dataset of post-stimulus epochs was employed to assess:

1. Braking reaction time (BRT): This analysis measures the time required to press the brake pedal once the guide vehicle's rear brake lights turned on (see Figure 3). BRT was computed for each emergency braking situation simply as the difference between the response time and the stimulus time.
2. EMG-based Leg movement (LEG): This analysis shows the right leg movement (at the muscular level) that is performed by the driver to press the brake pedal. LEG was computed as follows: (*i*) the EMG signal was high-pass filtered at of 10 Hz using a 2nd-order Chebyshev-type filter; (*ii*) the absolute value of the filtered signal was then computed; (iii) the Hilbert transform (Myers et al., 2003) was computed; (*iv*) the magnitude of the Hilbert transformed signal was used as the muscle-based leg movement.

### 2.7. Detection of emergency braking intention

The recognition of emergency braking intention from normal driving was assessed using time-domain features of the EEG signals and two different classification algorithms, Support Vector Machine (SVM) and Convolutional Neural Network (CNN). For this, pre-response and normal driving epochs were used.

#### 2.7.1. Feature extraction

Time-domain features of the EEG signals were computed to recognize emergency braking intention from normal driving. The EEG signal of each electrode (duration of 1.5 s) was divided in 10 consecutive intervals of 150 ms with no overlapping and the arithmetic average was computed for each interval. The values of all electrodes were used to construct a matrix of features (i.e., a 2D map) **X** ∈ ℝ^*M*×*N*^, or equivalently, they were concatenated to construct the feature vector **x** ∈ ℝ^(*M*·*N*)×1^, where *M* = 7 is the number of electrodes and *N* = 10 is number of time intervals. Features extracted from normal driving and pre-response epochs were labeled as *normaldrivinginstances* or −1 and *brakingintentioninstances* or +1, correspondingly. The total number of instances averaged across-all-participants was 890 ± 48. Note that the resulting datasets were balanced due to features were extracted from two different epochs of the data segments (i.e., Pre-response and Non-braking epochs).

#### 2.7.2. Classifiers

A Convolutional Neural Network (CNN) was employed to discriminate between emergency braking intention from normal driving. A CNN is a special type of supervised deep learning based classification algorithm (LeCun et al., 1989b, 2015; Goodfellow et al., 2016) that have demonstrated remarkable success in the classification of multidimensional images (Krizhevsky et al., 2012; Farabet et al., 2013; Szegedy et al., 2015) and it was used to some EEG studies with successful results (Ren and Wu, 2014; Schirrmeister et al., 2017; Tabar and Halici, 2017).

The architecture of a CNN is based on a stack of hidden layers named convolution and pooling, and a feed forward Artificial Neural Network (ANN), which together gradually transform an input map up to obtain class probabilities. The equation that describes the convolutional operation is:

(1) $$\begin{array}{r} S\left(i,j\right)=\left(I\times K\right)\left(i,j\right)=\underset{m}{\sum }\underset{n}{\sum }I\left(i+m,j+n\right)\cdot K\left(m,n\right)+b \end{array}$$

Where a kernel *K* of size *m* × *n* is convoluted (slided over the input map spatially) with input map of size *I*(*i, j*) and sum to bias *b* to construct output feature map *S*(*i, j*). Convolution and pooling layers are typically connected one by one with the aim of transforming an input map into many feature maps, thus, their effect is to perform an automatic feature extraction (LeCun et al., 1989a; LeCun and Bengio, 1998). In a CNN, the number of convolution layers, the number of kernels, the kernel's size, number of pooling layers, the pooling size, and the structure of the feed forward ANN are tunable parameters (also known hyperparameters), while the weights and bias in the kernels and in the feed forward ANN are parameters that are learned from a training set.

**Figure 4:**
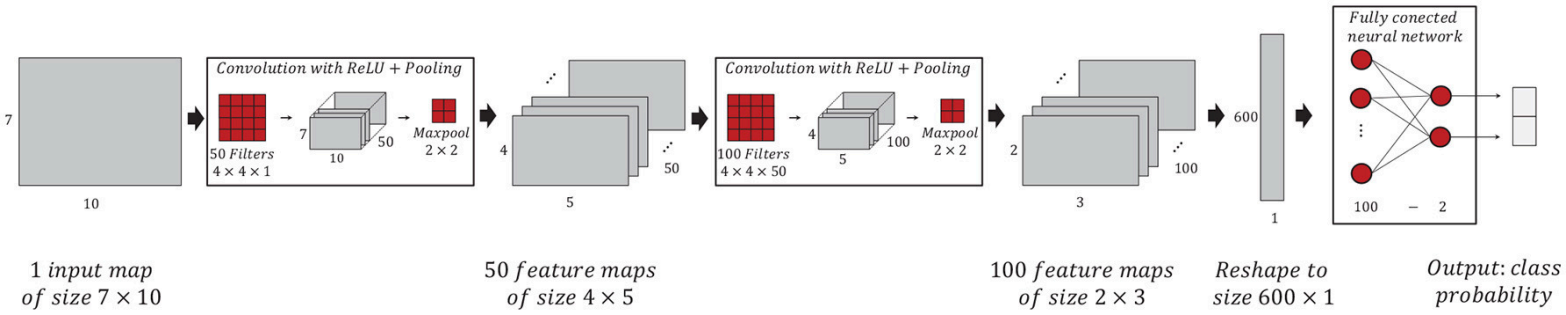
Illustration of the CNN algorithm implemented to discriminate between emergency braking intention from normal driving. The CNN consists of two pairs of convolution and pooling layers followed by a feed forward ANN.

The architecture of the CNN employed in this work is illustrated in Figure 4. It consists of two pairs of convolution and pooling layers followed by a feed forward ANN with a hidden layer (We tested several CNN architectures to find the one that would give us the best ratio of higher performance to lower compute time. For this, we tuned the hyperparameters: the number of CNN layers, the number of kernels, the number of epochs, and the size batch of training data). The input map size is *M* = 7 × *N* = 10, that is, 10 time domain features for each of the 7 electrodes. The first convolution-pooling pair consisted of *K* = 50 kernels of size 4 × 4, the rectified linear unit as activation function, maximum pooling with non-overlapping regions of size 2 × 2 and dropout technique was applied with 15% retention rate yielding to 720 estimated parameters corresponding 680 convolutional kernel weights, 50 convolutional kernel biases. The output map of this layer resulted in 50 feature maps of size 4 × 5 The second convolution-pooling pair consisted of *K* = 100 kernels of size 4 × 4 with the rectified linear unit as a activation function while the subsequent pooling also consisted of a maximum poling with non-overlapping regions of size 2 × 2 dropout technique was also applied with same retention rate yielding to 68100 parameters corresponding 68000 convolutional kernel weights, 100 convolutional kernel biases. The output map of this layer resulted in 100 feature maps of size 2 × 3. The feed forward ANN consisted of 100 input neurons and 2 neurons in the output layer. The activation function in the hidden layer is the sigmoid while in the output layer is the soft-max. Dropout was applied with with 85% retention rate. In total, ANN's layers yielding to 51187 parameters corresponding 51085 nodes weights, 102 biases. To sum up, this CNN architecture contains 120.017 learnable parameters.

In addition, we used a Support Vector Machine (SVM) for the classification since this algorithm has showed good performance in applications with EEG signals (Lotte et al., 2007; Vega et al., 2017). A support vector machine takes as input a set of *n* feature vectors $\vec{x_{i}}$ together with their labels *y*_*i*_ ∈ {1, −1}. The idea behind SVMs is to find the hyperplane that maximizes the distance between the examples of the two classes {1, −1}. This is done by finding a solution to the optimization problem

(2) $$\begin{array}{r} \text{min}\underset{\vec{w},b,\xi }{\;}C\overset{n}{\underset{i=1}{\sum }}\xi_{i}+\frac{1}{2}||\vec{w}|{|}^{2}, \end{array}$$

subject to the condition

(3) $$\begin{array}{r} y_{i}\left({\vec{w}}^{T}\phi \left(\vec{x_{i}}\right)+b\right)\geq 1-\xi_{i}, \end{array}$$

where $\vec{w}$ is the normal to the hyperplane, and ξ_*i*_ ≥ 0 are slack variables that measure the error in the misclassification of $\vec{x_{i}}$.

#### 2.7.3. Training

The implementation and training of both classification algorithms relied on the TensorFlow software library (Abadi et al., 2015). Given a training set, the algorithms were trained in 200 training steps following next instructions: (*i*) to initialize estimated parameters randomly (only for the training step 1); (*ii*) a batch data is sampled from training data (batch size is 20% of training data); (*iii*) classification model is fed with the batch data; (*iv*) obtaining the prediction outputs of the classification model; (*v*) comparison of the predicted outputs with the actual labels (to find the error trough a cost function); (*vi*) cost function optimizing; (*vii*) updating estimated parameters; and (*viii*) the rest of the training set was used to evaluate the model performance for each training step (validation test). The learning rate was set to 0.005 and the cross entropy was used as the cost function.

#### 2.7.4. Evaluation

The total recorded data was splitted in two mutually exclusive sets. The training set consisted of 75% of the data, and the evaluation set consisted of 25% of the data. The classifiers are trained using the training set and final classification is performed on the evaluation set. Performance metric was classification accuracy which was computed as:

(4) $$\begin{array}{r} accuracy=\frac{TP+TN}{TP+TN+FP+FN} \end{array}$$

where *TP* is the true positive rate, *TN* is the true negative rate, *FP* is the false positive rate, and *FN* is the false negative rate. This procedure is repeated 100 times, and *mean* ± *std* of the performance metrics were computed. Here we only reported results obtained in the final classification. However, validation and evaluation results during training are provided as Supplementary Material section.

The significant classification accuracy chance level was calculated with the binomial distribution (Combrisson and Jerbi, 2015) using the number of classes *N*_*clases*_ = 2, the minimum number of samples across all participants *N*_*samples*_ = 600 and a confidence level of α = 0.05. Consequently, the significant classification accuracy chance level is *accuracy*_*chance*_ = 53.6%. To examine significant differences between a distribution of accuracy and *accuracy*_*chance*_ the Wilcoxon signed-rank test was applied, while to examine significant differences between two distributions of accuracy the Wilcoxon rank-sum test was applied.

We estimated the receiver operating characteristic (ROC) graphs and area under ROC curve (AUC). ROC curve provides an optimal visualization of a classifier performance and allows to compare the performances between different classifiers. ROC curve is useful to show skewed class distribution and unequal classification error costs (Fawcett, 2006). ROC graphs are two-dimensional graphs in which true positive rate is plotted on the *y*-axis and false positive rate is plotted on the *x*-axis. A method to compare classifiers is to calculate the area under the ROC curve. AUC reduces ROC performance to a single scalar value representing expected performance. AUC is a value be between 0 and 1.0 due to it represents a portion of the area of the unit square. In addition, we reported the precision-recall graphs as other way to measure of success of prediction of the classifiers. These results are provided as Supplementary Material section.

## 3. Results

We first show an analysis of the braking reaction time under different cognitive states. Then we present results on the detection of braking intention under these different cognitive states which include stress, fatigue and workload.

### 3.1. Braking reaction time analysis

This section analyses the braking reaction time (BRT) under different cognitive states in order to assess how the reaction time is affected by stress, fatigue, and workload.

**Figure 5:**
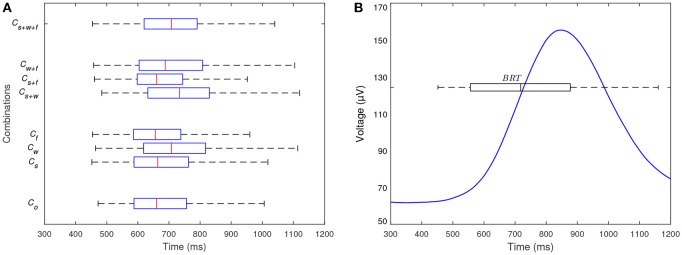
**(A)** Distributions of BRT across all participants for each experimental combination. At least one of the distributions is significantly different which indicates different BRT across the experimental combinations. **(B)** Time-resolved LEG signal averaged across all participants for all experimental combination. The distribution of BRT across all participants and experimental combination is also shown. The reference time *t* = 0 correspond to the stimulus. The LEG signal begins to increase at ~600 ms (i.e., prior to the median BRT) and peaks at ~850 ms.

The BRT averaged across-all-participants was 718 ± 162 ms (minimum 452 and maximum 1192 ms). This is congruent with previous studies that have reported BRT in the range of 720 and 1250 ms (Broen and Chiang, 1996; Green, 2000; Wasserman et al., 2017). In addition, Figure 5A shows the across all participants distributions of BRT independently for each experimental combination and this results are summarized in Table 2. When comparing these distributions all together, at least one is significantly different (*p* < 0.05, Kruskal-Wallis test), which indicates different BRT across the experimental combinations. To better explore the effect of stress, workload and fatigue on the BRT, the distribution of BRT in combination *C*_*o*_ (which does not include any factor) was individually compared with the other distributions (which include one, two, or three factors).

**Table 2:** Summary of BRT (units of ms) for the eight experimental combinations.

**Combination**	**Mean ± std**	**Max**	**Min**
*C*_*o*_	687 ± 138	1176	472
*C*_*s*_	689 ± 136	1192	452
*C*_*w*_	733 ± 154	1190	464
*C*_*f*_	676 ± 125	1176	454
*C*_*s*+*w*_	747 ± 151	1180	484
*C*_*s*+*f*_	681 ± 125	1192	460
*C*_*w*+*f*_	715 ± 145	1180	458
*C*_*s*+*w*+*f*_	724 ± 151	1180	484

*The average BRT is above 700 ms in experimental combinations that include workload*.

For the case of BRT with absence and presence of stress (*C*_*o*_ and *C*_*s*_), the median of BRT is slightly greater in presence of stress (664 ms) than in absence of it (660 ms), however, no significant differences are found between the two distributions (*p* > 0.05, Wilcoxon rank-sum test). This shows that although BRT is marginally higher in emergency braking situations with stress, these differences are not significant. This shows that the influence of stress increased BRT in 2%, however, this increment was not significant. For the case of BRT with absence and presence of fatigue (*C*_*o*_ and *C*_*f*_), the distributions present no significant differences (*p* > 0.05, Wilcoxon rank-sum test) and the medians are similar (660 and 656 ms for absence and presence of fatigue, respectively). Those results indicate that the presence of fatigue did not presented BRT significantly different than in the absence of it. The reason of why stress and fatigue did not resulted in different BRT may be due to the fact that participants did not perceive the high-intensity ambulance sound or the afternoon session as annoying, dangerous, and/or exhausting situations since the experiment was performed in a fully-controlled environment. This is consistent with the results obtained with the NASA-TLX index, where all participants expressed similar levels of mental, physical demand, effort, and frustration in the experiments carried out during in the afternoon and the experiments executed in the morning.

Nonetheless, for the case of BRT with absence and presence of workload (*C*_*o*_ and *C*_*w*_), the distributions are significant different (*p* < 0.05, Wilcoxon rank-sum test) and the median of BRT is greater in presence of workload (708 ms) than in absence of it (660 ms), which shows that the workload significantly increases the BRT. In this case, the BRT was 7% higher for emergency braking in the presence of workload than in the absent of it. This shows that the execution of a secondary task (as the workload was induced in our experiments) during emergency braking situations increases the time needed to press the brake pedal. The processing of additional cognitive and peripheral tasks required by the additional workload may explain this delayed response.

In addition, distributions of BRT that include two or three factors present significant differences (*p* < 0.05, Wilcoxon rank-sum test) and greater median than *C*_*o*_ solely when they include workload (i.e., *C*_*s*+*w*_, *C*_*w*+*f*_, and *C*_*s*+*w*+*f*_). Interestingly, the BRT of experimental combinations that include simultaneously stress, workload, and/or fatigue was also significantly different and higher only in combinations with presence of workload. This confirms the increased BRT due to the execution of a simultaneous task. To sum up, these analyses show that, in our experimental setting, the BRT does not increase in emergency braking situations that include stress and/or fatigue, however the BRT is significantly different and higher in emergency braking situations with workload.

The time-resolved LEG signal averaged across all participants is presented in Figure 5B. Note that the distribution of BRT across all participants and experimental combination is also displayed. The LEG signal is minimum and roughly constant during and a few milliseconds after the stimulus. Then, it starts to increase and at ~600 ms has increased by 10% with respect to its roughly contact value around the stimulus. The signal then peaks at ~850 ms. Finally, the signal gradually decreases up to its minimum and constant level. Note that the increase of the LEG signal initiates prior to 718 ms which is the across-all participants average BRT. This behavior is expected because it shows a muscular response previous to the mechanical deflection of the brake pedal.

### 3.2. Detection of emergency braking intention

In a first classification analysis we show results when participants experienced stress, workload, and/or fatigue while driving. We consider that this is a realistic driving situation, and in our experiments corresponds to gartering the data from all experimental combinations. In this analysis, we tested two individual classifiers for each participant following the procedure explained in section 2.7.4.

**Figure 6:**
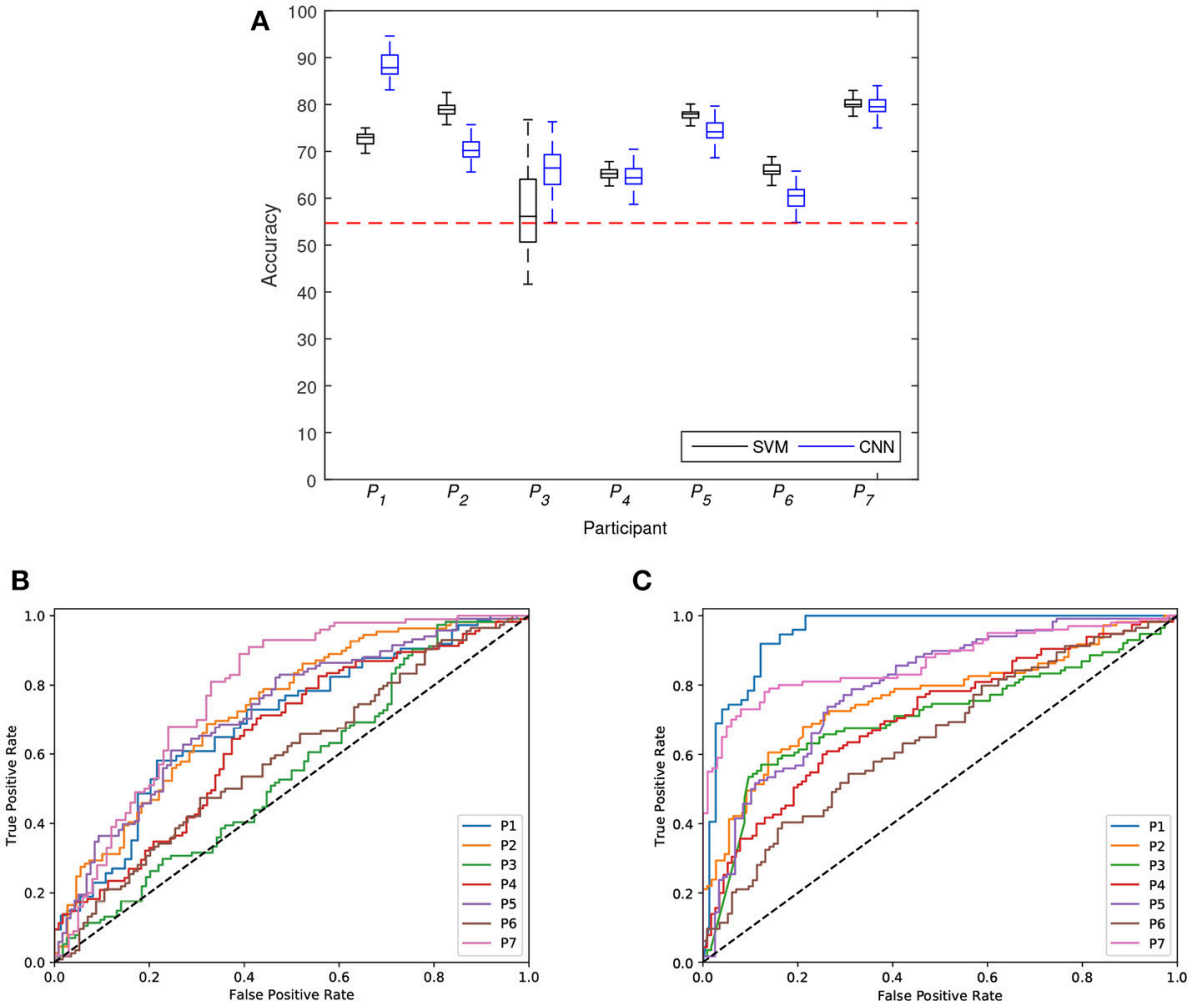
**(A)** Distribution of classification accuracy achieved with SVM and CNN for each participant (*P*_1_ to *P*_7_). The horizontal dotted red line represents the significant chance level or *accuracy*_*chance*_. **(B)** ROC curves for SVM classifiers for each participant. **(C)** ROC curves for CNN classifiers for each participant. The diagonal dotted black line represents the threshold *AUC* = 0.5.

Figure 6A shows the distributions of classification accuracy achieved with SVM and CNN for each participant (*P*_1_ to *P*_7_). Both classifiers in all participants (except for participant *P*_3_ with the SVM) presented distributions of classification accuracy that were significantly different and higher than *accuracy*_*chance*_ (*p* < 0.05, Wilcoxon signed-rank test). Participants *P*_1_ and *P*_3_ presented distributions of classification accuracy that were significant different and greater for the CNN than the SVM (*p* < 0.05, Wilcoxon rank-sum test). For participants *P*_2_ and *P*_6_, the SVM presented distributions of classification accuracy that were significant different and greater than the CNN (*p* < 0.05, Wilcoxon rank-sum test). Finally, no significant differences were found between the medians of the classification accuracy distributions of the SVM and CNN (*p* > 0.05, Wilcoxon rank-sum test) in participants *P*_4_, *P*_5_, and *P*_7_. Figures 6B,C shows the ROC curves created by thresholding a test set for SVM and CNN for each participant (*P*_1_ to *P*_7_). Both classifiers in all participants presented curves above the threshold. These results are also summarized in Table 3. The *mean* ± *std* of participants *P*_1_ and *P*_3_ was greater for the CNN (88.1 ± 2.7 and 65.2 ± 6.5, respectively) than for the SVM (72.7 ± 1.3 and 57.3 ± 8.8, respectively). On the contrary, the *mean* ± *std* of classification accuracy in participants *P*_2_ and *P*_6_ was greater for the SVM (78.9 ± 1.2 and 60.0 ± 1.3, respectively) than for the CNN (70.5 ± 2.5 and 60.2 ± 2.3 respectively). The remaining participants, *P*_4_, *P*_5_, and *P*_7_, presented similar average accuracy in both classifiers (65.0 ± 1.5, 77.7 ± 1.2, and 80.1 ± 1.3, respectively for the SVM, and 64.7 ± 2.6, 74.4 ± 2.6, and 79.7 ± 2.1, respectively for the CNN). Note that in all cases, the average classification accuracy is above *accuracy*_*chance*_. Finally, the grand-average across-all-participants is similar for the two classifiers, 71.10 ± 8.69 for the SVM and 71.83 ± 9.60 for the CNN. To compare the performance of the ROC curve of the two classifiers, the AUC score was obtained for each participant and reported in the Table 3. SVM classifiers presented values above 0.6 in all participants (except for *P*_3_ and *P*_6_) which is considered a discriminatory standard with AUC average of 0.66. CNN presented values above 0.6 in all participants with AUC average of 0.77. To sum up, these results show significant recognition of emergency braking intention irrespective of the fact that the emergency situations were performed while driver's experienced or not cognitive factors as stress, workload, and fatigue.

**Table 3:** Summary of classification accuracy results for each participant (*P*_1_ to *P*_7_) achieved with the SVM and CNN.

**Participant**	**SVM**				**CNN**			
	**Mean ± std**	**Max**	**Min**	**AUC**	**Mean ± std**	**Max**	**Min**	**AUC**
*P*_1_	72.7 ± 1.3	75.0	69.6	0.69	88.1 ± 2.7	94.6	78.4	0.94
*P*_2_	78.9 ± 1.2	82.6	75.7	0.73	70.5 ± 2.5	78.4	65.6	0.77
*P*_3_	57.3 ± 8.8	76.8	41.7	0.55	65.2 ± 6.5	79.0	48.7	0.68
*P*_4_	65.0 ± 1.5	69.1	60.0	0.65	64.7 ± 2.6	70.4	58.7	0.71
*P*_5_	77.7 ± 1.2	80.5	73.7	0.72	74.4 ± 2.6	82.2	68.6	0.82
*P*_6_	66.0 ± 1.3	68.9	62.7	0.59	60.2 ± 2.3	65.8	54.8	0.67
*P*_7_	80.1 ± 1.3	83.0	76.5	0.78	79.7 ± 2.1	84.0	74.5	0.87
*Average*	71.1 ± 8.7	83.0	41.7	0.67	71.8 ± 9.6	94.6	48.7	0.77

*The lower row shows the grand-average across-all-participants*.

In a second classification analysis, the detection of emergency braking intention was assessed by gathering the data from all participants (training data size = 4674 and test data size = 1556). The classification accuracy was on average 54.5 ± 0.9 for the SVM, while it was 68.4 ± 1.3 for the CNN. On the one hand, these results show that the accuracy is 13.9% greater for CNN than SVM. On the other hand, the accuracies are lower than when using the data of each participant individually (the grand-average across-all-participants was 71.1 ± 8.7 and 71.8 ± 9.6 for the SVM and CNN, respectively). Indeed, the use of the data from all participants reduces accuracy in 16.6 and 3.5% for the SVM and CNN, respectively. Note that the across-participants driving patterns discrepancies and differences in the brain signatures may explain this different performance.

**Figure 7:**
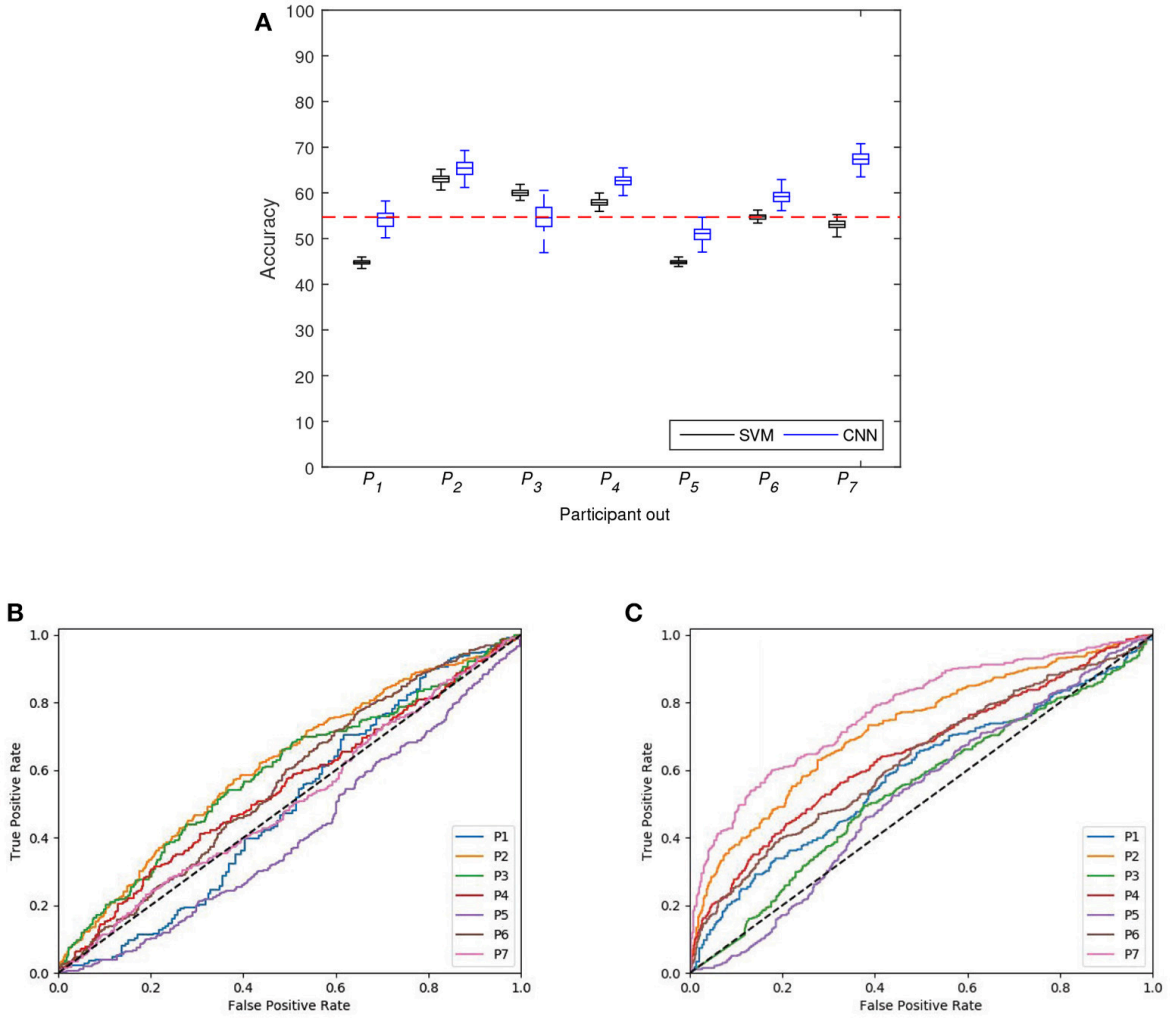
**(A)** Distribution of classification accuracy achieved with SVM and CNN for the classification experiment leave-one-out participant. The horizontal dotted red line represents the significant chance level or *accuracy*_*chance*_. **(B)** ROC curves for SVM classifiers for each leave-one-out participant. **(C)** ROC curves for CNN classifiers for leave-one-out participant.

In the third classification analysis we assessed the detection of emergency braking intention for each of the seven participants by training a classifier exclusively with data from the remaining six participants. With this experiment we study the transferability of a learned classifier in order to detect emergency braking in a completely new participant. The final goal would be to train a classifier with available participants in order to be used by a new driver. Figure 7A shows the distributions of classification accuracy for this leave-one-out participant classification experiment for the two classifiers. The results are also summarized in Table 4. The CNN presented distributions of classification accuracy that were significantly different and higher than *accuracy*_*chance*_ (*p* < 0.05, Wilcoxon signed-rank test) in four out of the seven participants (*P*_2_, *P*_4_, *P*_6_, and *P*_7_), while the SVM presented distributions of classification accuracy that were significantly different and higher than *accuracy*_*chance*_ (*p* < 0.05, Wilcoxon signed-rank test) in three out of the seven participants (*P*_2_, *P*_3_, and *P*_4_). In addition, the classification accuracy was on average 54.1 ± 6.7 for the SVM, while it was 59.2 ± 6.0 for the CNN. These results show higher classification accuracies for CNN than SVM, however they are lower than in the case of learning a user-specific classifier (grand-average across-all-participants of 71.1 ± 8.7 and 71.8 ± 9.6 for SVM and CNN, respectively) and in the case of using of the data from all participants (average of 54.5 ± 0.9 and 68.4 ± 1.3 for SVM and CNN, respectively). Figures 7B,C shows the ROC curves created by thresholding a test set for SVM and CNN for each leave-one-out participant classification. Both classifiers presented all ROC curves above the threshold (except for SVM in *leave*−*one*−*outparticipant* 1 and 5 and for CNN in *leave*−*one*−*outparticipant* 5). To compare the performance of the ROC curve of the two classifiers, the AUC score was obtained for each leave-one-out participant and reported in the Table 4. SVM classifiers presented values above 0.6 only for *P*_2_. CNN presented values above 0.6 in three leave-one-out participants (*P*_2_, *P*_4_, and *P*_7_). To sum up, both classifiers have similar accuracy values. However, CNN presented higher AUC values than SVM which indicates that CNN was more sensitive in predicting the braking intention than SVM classifier. The transferability results show a lower performance than personalized individual classifiers. From our point of view this makes sense since different people deal in different ways with different cognitive states. For example, previous works on stress detection show also better performance on personalized individual classifiers (Mozos et al., 2017). However, an increment on the number and diversity of participants may improve the transferability results.

**Table 4:** Summary of classification accuracy results achieved with SVM and CNN in the classification experiment leave-one-out participant.

**Participant**	**SVM**				**CNN**			
**out**	**Mean ± std**	**Max**	**Min**	**AUC**	**Mean ± std**	**Max**	**Min**	**AUC**
*P*_1_	44.7 ± 0.6	46.0	43.3	0.44	54.2 ± 1.9	58.2	50.2	0.54
*P*_2_	63.1 ± 1.1	65.2	59.7	0.62	65.3 ± 1.8	69.3	58.1	0.74
*P*_3_	60.0 ± 0.8	61.9	58.4	0.59	54.6 ± 3.1	60.5	46.9	0.57
*P*_4_	57.9 ± 0.9	60.0	55.2	0.57	62.7 ± 1.4	66.2	59.2	0.65
*P*_5_	44.8 ± 0.5	46.2	42.9	0.47	50.9 ± 1.7	56.3	47.0	0.50
*P*_6_	54.7 ± 0.7	56.2	52.8	0.53	59.2 ± 1.5	63.3	55.0	0.61
*P*_7_	53.3 ± 1.2	57.0	50.4	0.54	67.2 ± 1.7	70.8	60.5	0.74
*Average*	54.1 ± 6.7	65.2	42.9	0.53	59.2 ± 6.0	70.8	46.9	0.62

*The lower row shows the grand-average across-all-participants*.

## 4. Discussion and conclusion

This work shows the feasibility of using driver's electroencephalographic (EEG) signals to recognize the intention to perform an emergency braking in driving conditions where the drivers experienced realistic cognitive states such as stress, workload, and fatigue. The contribution of this paper is two-fold. First, our classification results indicate the possibility of detecting with high probability the emergency braking intention, which precedes the moment at which the mechanical activation of the brake pedal becomes observable. This can be used to incorporate this detection into driving assistance systems in order to avoid accidents by, for example, reducing the final braking reaction time to gain braking distance, or by maneuvering the car automatically in a sudden situation. Second, this works considers cognitive states that may negatively affect the driving. In particular we carried out experiments including stress, workload, and fatigue during the driving task. Notice that this work considers the early detection of braking intention form EEG irrespective of the cognitive state(s) experienced by the drivers. For this reason, we lumped the data of all cognitive states to carry out the classification between braking intention and normal driving. This is a novelty in our work as we are considering that the drivers experience cognitive states during driving rather than being driving in fully controlled and relaxed environment. The presented classification results provide evidence of the possibility of using the brain signals to anticipate the braking action despite the driver is experiencing typical cognitive factors that are known to negatively affect driving performance. The proposed CNN provided significant classification between emergency braking intention from normal driving. It is not possible however to compare these results with previous studies due to those related works were performed in different experimental settings without contemplating the potential effect on brain processes of different driver's cognitive states cause by fatigue, stress, workload during driving a car. It is important to say that the classification accuracy rates presented herein and the cognitive states may differ in real driving situations, thus, more research is still needed to assess the detection of braking intention during realistic driving environments. A difficulty here would be to avoid the induction of cognitive states to consider real stress, workload, and fatigue during driving. In addition, execution of experiments in real driving situations should consider critical safety procedures that are not present in simulated environments. Regarding the computational algorithm required to recognize between braking intention and normal driving, it is important to consider that an extended set of instances is advantageous; however, this requires longer experiments to record more braking situations. Finally, it is also required to study the effect of artifacts as blinking, head movements, limb movements, etc., which will be embedded in the recorded EEG signals during real driving, since they may spoil the early detection of braking intention. The future direction in this research involves, first, to estimate the total time prior to the activation of the mechanical brake pedal that can be saved, as this time define the maximum gained braking distance, and second, to validate our system in real driving scenario to assess whether realistic conditions may affect the signatures in the brain signal that allow the detection of emergency braking intention (Martínez et al., 2018). In addition, we will extend the diversity of the participants by including people from different gender and age groups.

## Author contributions

JA and LH conceived and designed the experiments. LH performed the experiments. JA, LH, and OM analyzed the data. All authors participated in writing the document.

### Conflict of interest statement

The authors declare that the research was conducted in the absence of any commercial or financial relationships that could be construed as a potential conflict of interest.
