# Supplementary material for: EEG-Based Detection of Braking Intention Under Different Car Driving Conditions
Source: Front Neuroinform. 2018 May 29;12:29. doi: 10.3389/fninf.2018.00029 (PMC5992396; doi:10.3389/fninf.2018.00029)
Supplement: Supplementary file 1 [file Image_1.PDF]

Supplementary Material:
EEG-based detection of braking intention under different car driving conditions

1 SUPPLEMENTARY FIGURES

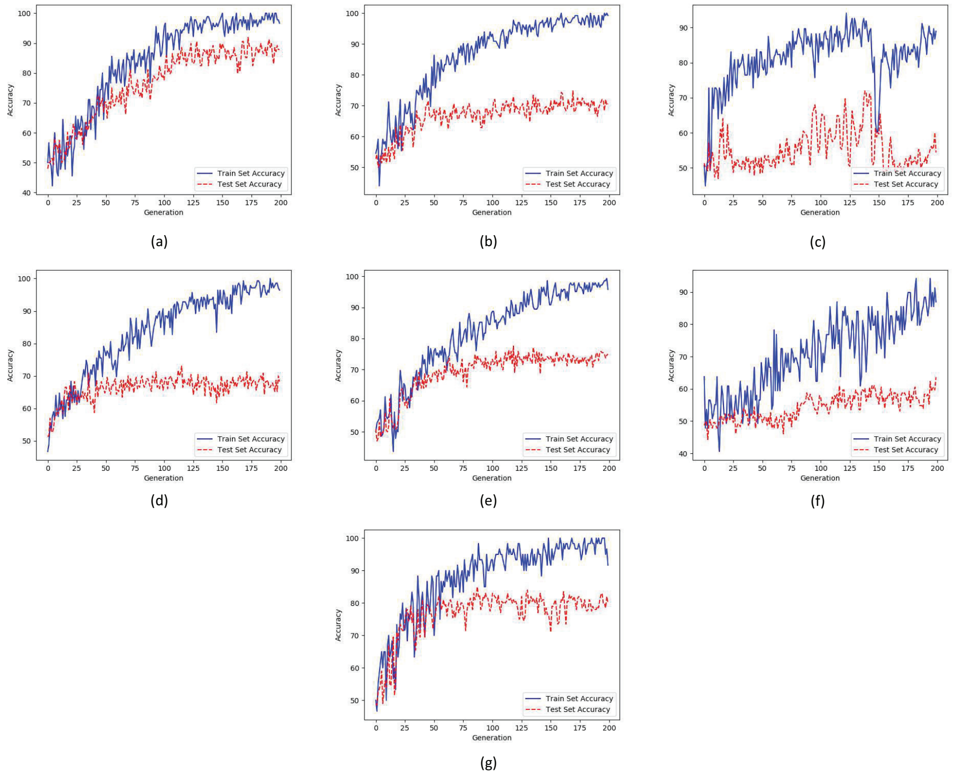

Figure S1. Illustration of classification accuracies in the train (blue curve) and test (red curve) sets during training epochs across all participants in the proposed CNN model to to discriminate between emergency braking intention from normal driving. Note that these results shows that the accuracy of both, train and test sets, increases with the number of epochs up to a steady state while no overfitting in the train set or drop in the test set is observed.

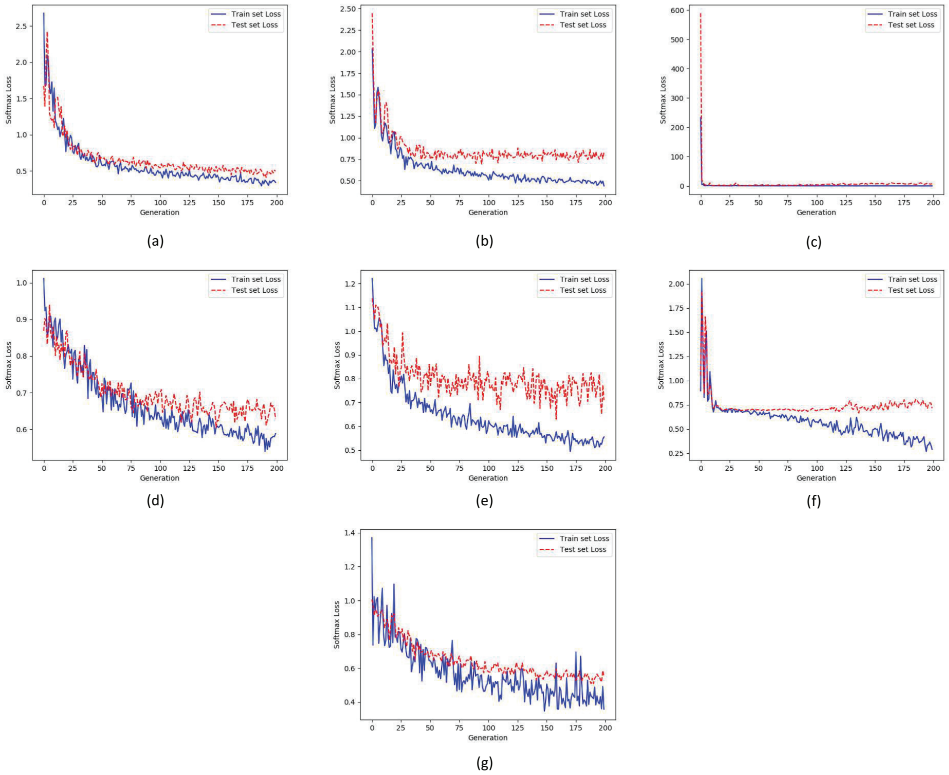

Figure S2. Illustration of the loss in the train (blue curve) and test (red curve) sets during training epochs for across-all-participants in the proposed CNN model to discriminate between emergency braking intention from normal driving. Note that these results shows that the loss of both, train and test sets, decreases with the same tendency throughout epochs.

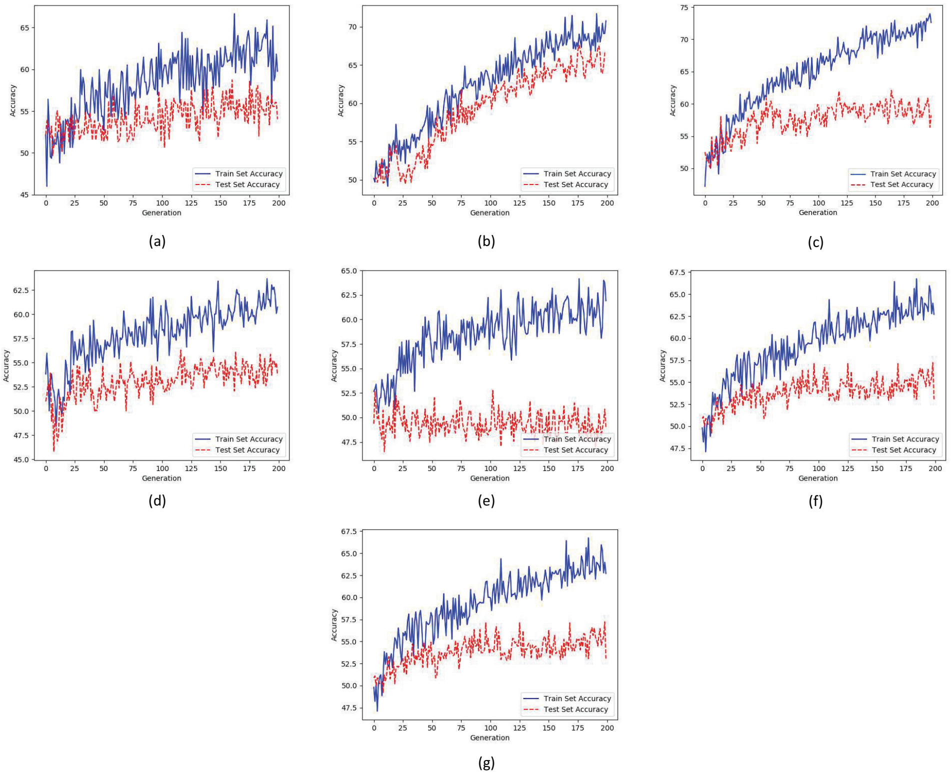

Figure S3. Illustration of classification accuracies in the train (blue curve) and test (red curve) sets during training epochs across leave-one-out participants in the proposed CNN model to discriminate between emergency braking intention from normal driving. Note that these results shows that the accuracy of both, train and test sets, increases with the number of epochs up to a steady state while no overfitting in the train set or drop in the test set is observed.

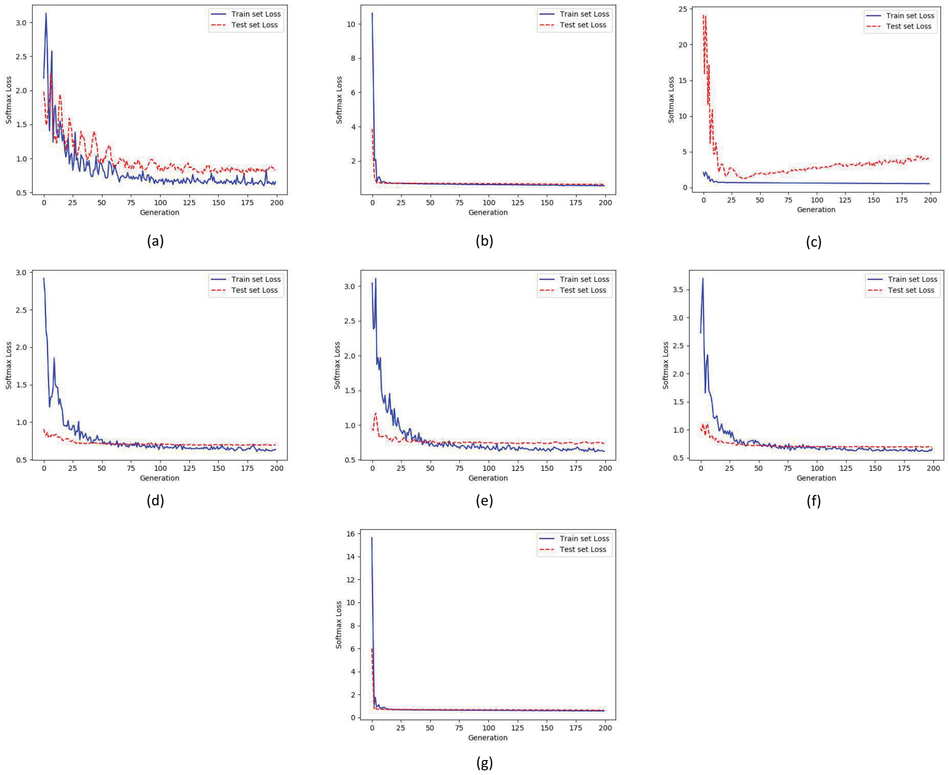

Figure S4. Illustration of loss in the train (blue curve) and test (red curve) sets during training epochs for across leave-one-out participants in the proposed CNN model to to discriminate between emergency braking intention from normal driving. Note that these results shows that the loss of both, train and test sets, decreases throughout epochs up to converge. In addition, note in figures b, c, d, f and g that loss converges quite early for both sets. A similar behavior is shown by the precision-recall curves in figures s5 b and d. This may indicate a fast learning rate when CNNs are applied to our specific dataset.

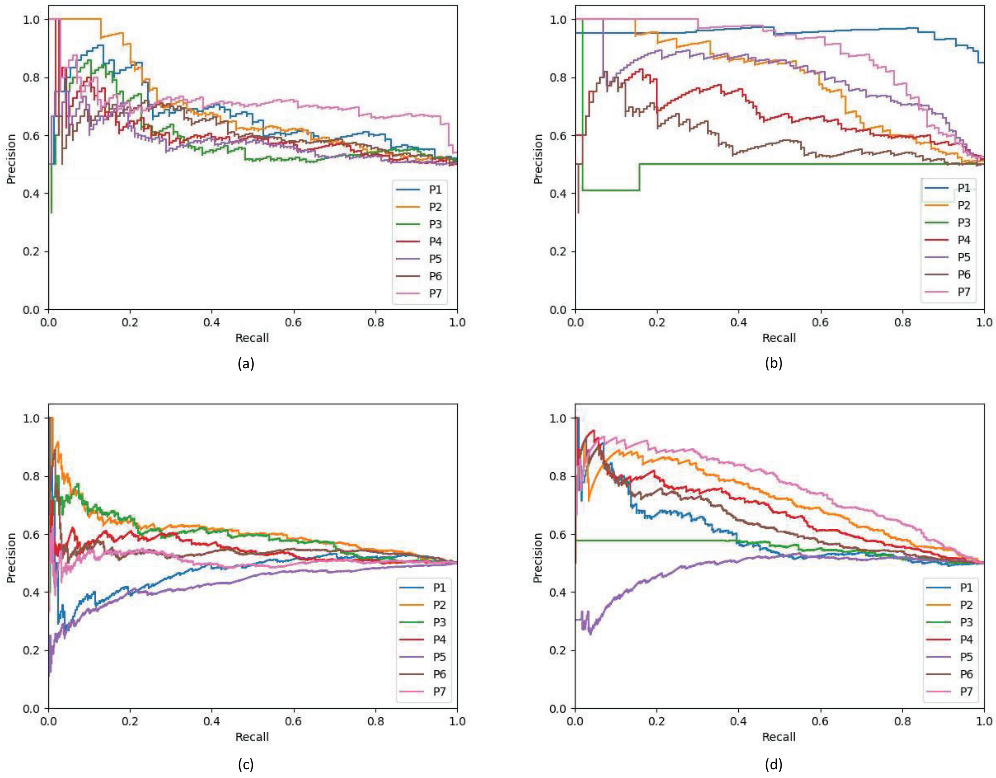

Figure S5. Plots of (a) Precision-Recall curves for SVM classifiers for each participant. (b) Precision-Recall curves for CNN classifiers for each participant. (c) Precision-Recall curves for SVM classifiers for each leave-one-out participant. (d) Precision-Recall curves for CNN classifiers for each leave-one-out participant.

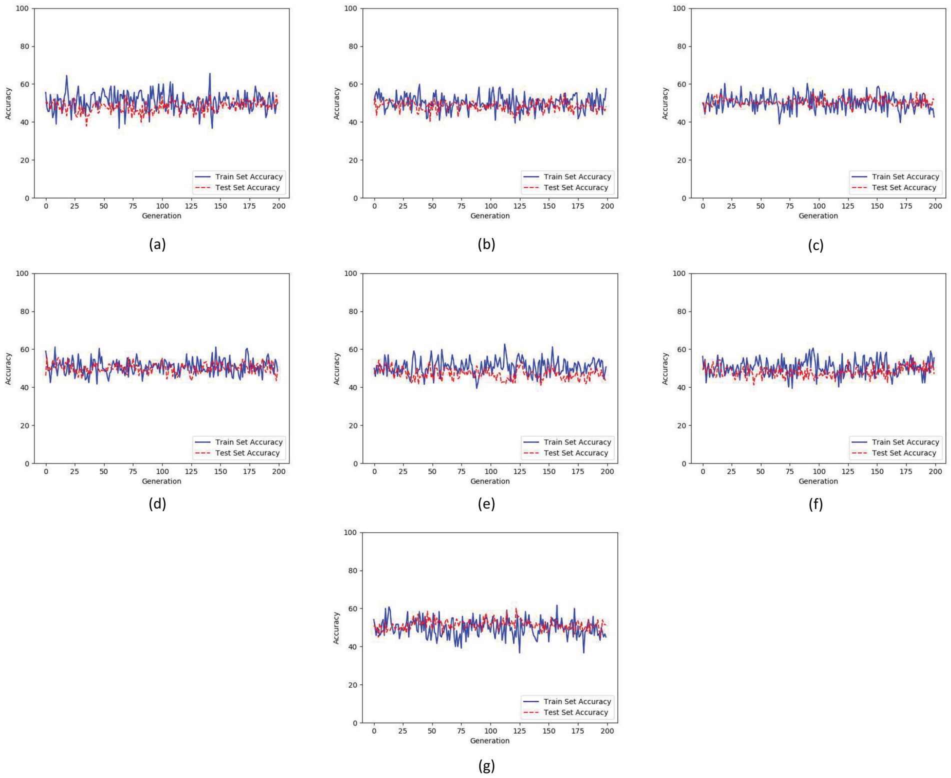

Figure S6. Accuracies in the train set (red curve) and test set (blue curve) across epochs using shuffle labels for the participant classification scenario. These results shows that in all participants, the accuracy is fluctuating around the theoretical chance level (50%) and not increment is observed as the epochs increases.

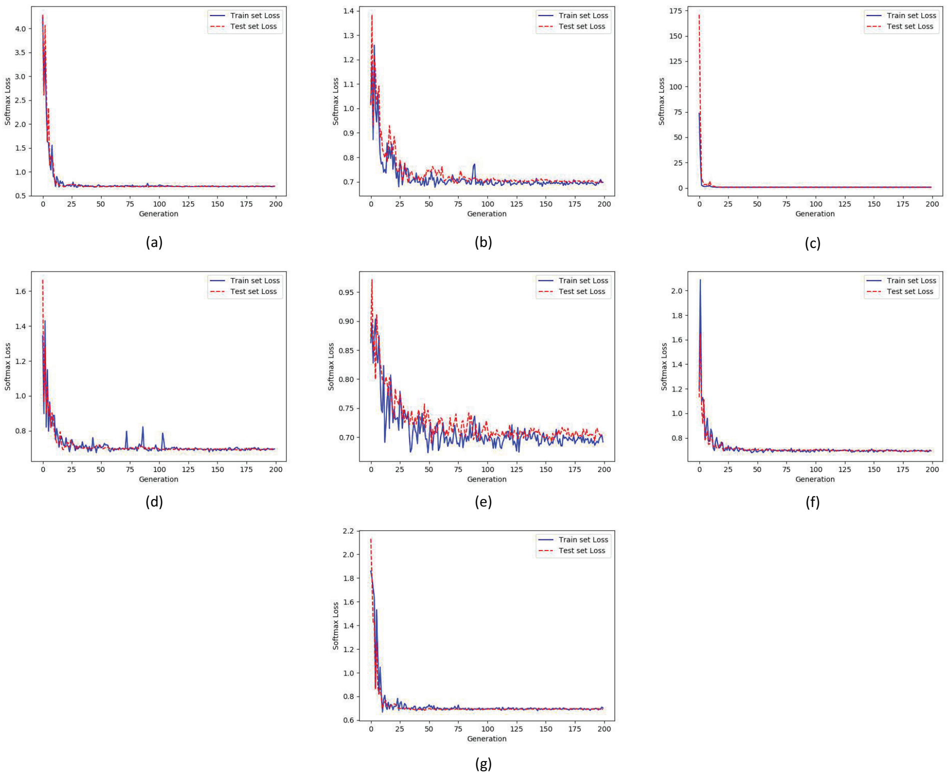

Figure S7. Loss in both train (red curve) and test (blue curve) sets using shuffle labels for the participant classification scenario. These results show a decrease in the loss through the first epochs up to reaching a steady value. This is due to the fact that the CNN model predictions give similar probability values for each of the classes such that the cost function minimizes the loss. The classification model seeks to reduce the loss as much as possible, which is achieved by assigning the same probability to the different classes. This situation has the effect that the classifiers accuracy is at the chance level as shown in figure S6.

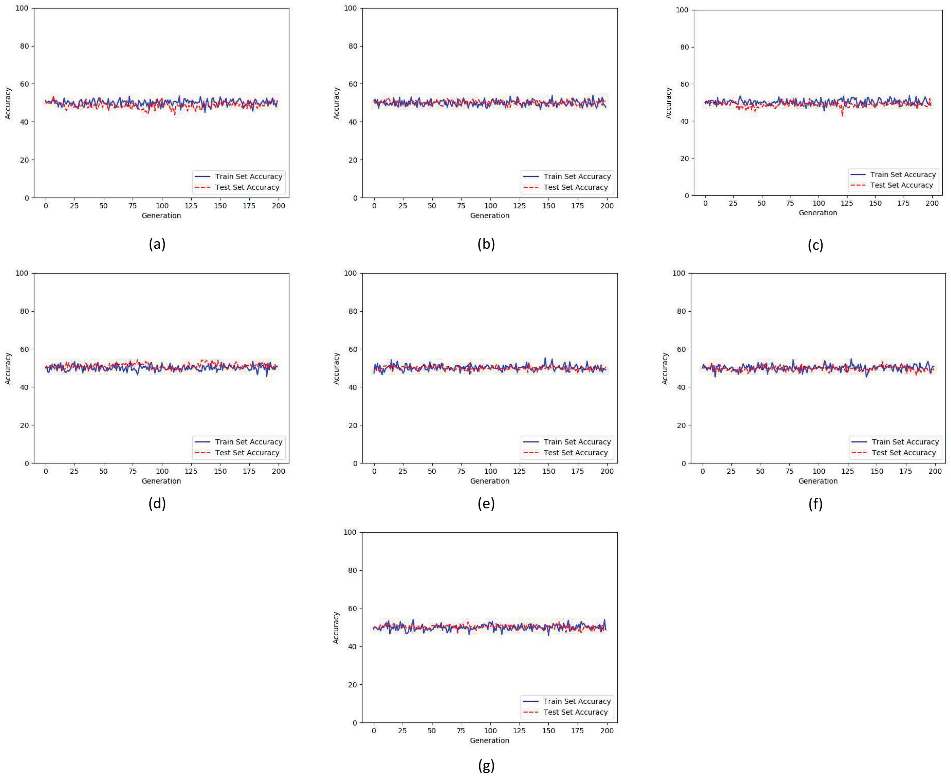

Figure S8. Accuracies in the train set (red curve) and test set (blue curve) across epochs using shuffle labels for the leave one out participant classification scenario. These results shows that in all participants, the accuracy is fluctuating around the theoretical chance level (50%) and not increment is observed as the epochs increases.

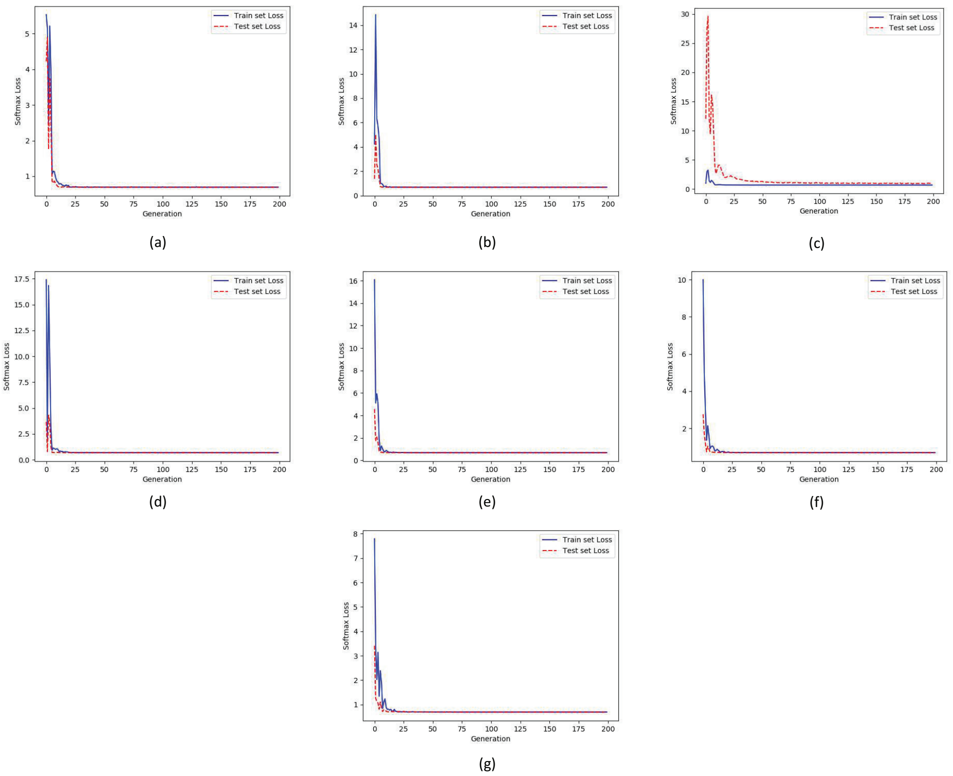

Figure S9. Loss in both train (red curve) and test (blue curve) sets using shuffle labels for the leave one out participant classification scenario. These results show a decrease in the loss through the first epochs up to reaching a steady value. This is due to the fact that the CNN model predictions give similar probability values for each of the classes such that the cost function minimizes the loss. The classification model seeks to reduce the loss as much as possible, which is achieved by assigning the same probability to the different classes. This situation has the effect that the classifiers accuracy is at the chance level as shown in figure S8.
